# Ferric Ammonium Citrate Reduces Claudin-5 Abundance and Function in Primary Mouse Brain Endothelial Cells

**DOI:** 10.1007/s11095-025-03826-2

**Published:** 2025-02-12

**Authors:** Pranav Runwal, Jae Pyun, Stephanie A. Newman, Celeste Mawal, Ashley I. Bush, Liam M. Koehn, Joseph A. Nicolazzo

**Affiliations:** 1 Drug Delivery, Disposition and Dynamics, Monash Institute of Pharmaceutical Sciences, Monash University, Parkville, VIC Australia; 2 Oxidation Biology Lab, Melbourne Dementia Research Centre, Florey Institute of Neuroscience and Mental Health, University of Melbourne, Parkville, VIC Australia; 3 University of Melbourne, Parkville, VIC Australia

**Keywords:** blood–brain barrier, breast cancer resistance protein, claudin-5, iron overload, P-glycoprotein

## Abstract

**Background:**

Iron overload is implicated in many neurodegenerative diseases, where there is also blood–brain barrier (BBB) dysfunction. As there is a growing interest in the role of iron in modulating key BBB proteins, this study assessed the effect of iron on the expression and function of P-glycoprotein (P-gp), breast cancer resistance protein (BCRP) and claudin-5 in primary mouse brain endothelial cells (MBECs) and their abundance in mouse brain microvessel-enriched membrane fractions (MVEFs).

**Methods:**

Following a 48 h treatment with ferric ammonium citrate (FAC, 250 µM), MBEC protein abundance (P-gp, BCRP and claudin-5) and mRNA (*abcb1a*, *abcg2*, and *cldn5)* were assessed by western blotting and RT-qPCR, respectively. Protein function was evaluated by assessing transport of substrates ^3^H-digoxin (P-gp), ^3^H-prazosin (BCRP) and ^14^C-sucrose (paracellular permeability). C57BL/6 mice received iron dextran (100 mg/kg, intraperitoneally) over 4 weeks, and MVEF protein abundance and iron levels (in MVEFs and plasma) were quantified via western blotting and inductively coupled plasma-mass spectrometry (ICP-MS), respectively.

**Results:**

FAC treatment reduced P-gp protein by 50% and *abcb1a* mRNA by 43%, without affecting ^3^H-digoxin transport. FAC did not alter BCRP protein or function, but decreased *abcg2* mRNA by 59%. FAC reduced claudin-5 protein and *cldn5* mRNA by 65% and 70%, respectively, resulting in a 200% increase in ^14^C-sucrose permeability. *In vivo*, iron dextran treatment significantly elevated plasma iron levels (2.2-fold) but did not affect brain MVEF iron content or alter P-gp, BCRP or claudin-5 protein abundance.

**Conclusions:**

Iron overload modulates BBB transporters and junction proteins *in vitro*, highlighting potential implications for CNS drug delivery in neurodegenerative diseases.

## Introduction

Iron is one of the most ubiquitous biometals in the body, essential for human physiology due to its redox potential, which allows it to donate and accept electrons and drive energy production [1, 2]. This redox activity underpins numerous biological processes, including DNA synthesis and cellular metabolism [1, 2]. In the brain, the cycling of iron between ferric and ferrous states is particularly critical, supporting functions such as neurotransmitter synthesis, axonal growth, mitochondrial respiration, and signal transduction [3, 4]. However, this same chemistry predisposes iron to form reactive oxygen species, posing a risk of oxidative stress and potential cellular damage [2]. Maintaining iron homeostasis is essential for physiological stability in the brain, without which, there can be disruptions to cellular processes contributing to neurodegenerative conditions, and also complicating therapeutic delivery and efficacy [5, 6].

Post-mortem studies in Parkinson’s disease reveal a 255% increase in intracellular iron, where excessive iron-induced oxidative stress leads to dopaminergic neuron toxicity, and in Alzheimer’s disease, iron-driven free radicals exacerbate amyloid-beta plaques and neurofibrillary tangles, accelerating cognitive decline [7–10]. Another critical aspect of neurodegenerative diseases is disturbance to the blood–brain barrier (BBB), with evidence suggesting that iron accumulation may contribute to damage within brain endothelial cells, which form the BBB [5, 11, 12].

The BBB, a critical component of the neurovascular unit, separates the peripheral circulation from the central nervous system (CNS) [11, 13]. It is characterized by specialized endothelial cells which express tight junction (TJ) proteins, such as claudin-5, occludin, and zonula occludens-1, that mechanically connect the brain endothelial cells, thus limiting paracellular diffusion of molecules between the blood and brain [13]. The importance of TJ proteins is highlighted through observations in claudin-5 deficient mice, which exhibit increased BBB paracellular permeability, rapid brain extravasation of peripherally-circulating molecules, and ultimately death [14]. In addition to this physical barrier, efflux transporters expressed at the luminal membrane of brain endothelial cells limit the transcellular transfer of lipid soluble molecules from the blood into the brain and assist in the removal of exogenous compounds from the brain into the blood [15]. Efflux transporters, such as P-glycoprotein (P-gp) and breast cancer resistance protein (BCRP), are crucial for limiting CNS xenobiotic exposure by facilitating the extrusion of drugs and toxins from brain microvascular endothelial cells back into the blood [16, 17]. Dysfunction of these transporters has been shown to increase brain sensitivity to neurotoxins and to impair the clearance of endogenous toxins [18, 19]. Therefore, any modifications to the expression or function of TJs and efflux transporters can endanger neuronal health and increase CNS exposure to toxins, including drugs [20, 21]. This is also to be considered in neurodegenerative diseases given that altered brain microvascular P-gp and TJ functionality has been reported in Alzheimer’s disease and Parkinson’s disease i.e. diseases with brain iron accumulation [22–25], and these modifications could have possible implications on the CNS exposure of medicines.

Iron has previously been shown to elicit deleterious effects to the BBB and therefore could contribute to the above-mentioned modifications in BBB function that have been observed in Alzheimer’s disease and Parkinson’s disease [23, 25–28]. Recent studies have demonstrated that iron overload, induced by ferric ammonium citrate (FAC), a pH stable and cell permeable form of iron, downregulates P-gp protein and mRNA in immortalized human brain endothelial (hCMEC/D3) cells [27]. A similar study in the same cell line reported that FAC reduces mRNA expression, but not protein abundance, of BCRP [28]. However, because these studies were conducted in immortalized cells, responsiveness to stimuli might have been affected by their underlying genetic modification, potentially limiting the translation of gene changes into protein abundance. Therefore, studies in alternative *in vitro* models of the BBB, which are not limited by genetic manipulation required for immortalization, are needed to ensure the reported effects of iron are reproducible in more physiologically relevant systems. Furthermore, given that the overall functionality of the BBB is a result of an interplay between various cell types (including pericytes, astrocytes, and brain endothelial cells), it is imperative to investigate the effect of iron overload on efflux transporters and tight junctions *in vivo*.

The purpose of this study, therefore, was to investigate the effect of FAC-induced iron overload on the protein abundance of P-gp, BCRP, and claudin-5 in primary mouse brain endothelial cells (MBECs). The bi-directional transport of ^3^H-digoxin (to assess P-gp function), ^3^H-prazosin (to assess BCRP function) and ^14^C-sucrose (to assess TJ protein function) was assessed in these primary MBECs with and without FAC treatment. Additionally, abundance of these barrier proteins, as well as iron concentrations, were evaluated in brain microvessels isolated from C57BL/6 mice dosed with and without iron dextran, thus providing a comprehensive analysis of iron overload in models more closely resembling the BBB. The outcomes of this study provide novel insights into the impact of iron overload on the access of drugs, toxins and endogenous molecules into the CNS.

## Materials And Methods

### Materials

FAC, 4-(2-hydroxyethyl)−1-piperazineethanesulfonic acid (HEPES), dimethyl sulfoxide (DMSO), penicillin–streptomycin, Tween-20, sodium dodecyl sulphate (SDS), MTT(3-(4,5-dimethyl-2-thiazolyl)−2,5-diphenyl-2H-tetrazolium bromide), absolute ethanol, 2-mercaptoethanol, bovine serum albumin (BSA), Trypan Blue solution, iron dextran (100 mg/ml), human collagen Type IV and fibronectin were purchased from Sigma-Aldrich (St. Louis, MO). Dulbecco’s phosphate-buffered saline (D-PBS), Dulbecco’s modified eagle medium GlutaMAX™, Hanks balanced salt solution (HBSS), Gibco™ Sodium Pyruvate (100 mM) and heat-inactivated foetal bovine serum (FBS) were purchased from Life Technologies (Carlsbad, CA). ^3^H-digoxin, ^3^H-prazosin and ^14^C-sucrose were purchased from American Radiolabeled Chemicals (St Louis, MO). Ultima Gold™ liquid scintillation cocktail, and scintillation vials were purchased from Perkin-Elmer Life Sciences (Waltham, MA). Milli-Q water was obtained from a Millipore system (Billerica, MA). UltraPure™ distilled water, puromycin dihydrochloride, Pierce® IP lysis buffer, Mem-PER™ Plus Membrane Protein Extraction kit, constituting cell permeabilization and membrane solubilisation buffer, was purchased from Thermo Fisher Scientific (Waltham, MA). Complete Mini Protease Inhibitor Cocktail tablet was purchased from Roche (Basel, Switzerland).

#### Methods

### Isolation and Culturing of Primary MBECs

All animal experiments were undertaken following approval from the Monash Institute of Pharmaceutical Sciences Animal Ethics Committee (Ethics #34607) and performed in accordance with the National Health and Medical Research Council Guidelines for the care and use of animals for scientific purposes. Brains of C57BL/6 mice (6–8 weeks) were removed under sterile conditions and washed with cold HBSS buffer containing 0.33 mM pyruvate, hereafter referred by HBSS-S. MBECs were isolated using the Adult Brain Dissociation kit (Miltenyi Biotech, Germany). Meninges and outer blood vessels were removed by rolling brain on filter paper (11 μm pore size), and the cortex was isolated and transferred into a C-tube with enzyme mix 1 (enzyme P and buffer Z) prepared as per the manufacturer’s protocol. The cortex was gently homogenized, and enzyme mix 2 (Enzyme A and buffer Y) was added, also prepared according to the manufacturer’s protocol, before inserting the C-tube into a gentleMACS™ Octo Dissociator for 30 min (Miltenyi Biotech, Germany). The homogenate was centrifuged at 300 xg for 30 s, strained through a 70 μm MACS smart strainer, and washed with HBSS-S. The cell suspension was centrifuged at 300 xg for 10 min at 4 °C, the supernatant aspirated, and the pellet resuspended in HBSS-S and cold debris removal solution, prepared as per the manufacturer’s protocol. This suspension was overlaid with 4 mL of HBSS-S and centrifuged at 3000 xg for 10 min at 4 °C. The top two layers were aspirated, and the suspension was made up to 15 mL with HBSS-S. After gentle inversion and centrifugation at 1000 xg for 10 min at 4 °C, the pellet was resuspended in 1 mL of 1 × Red Blood Cell Removal solution (Miltenyi Biotech, Germany), incubated for 10 min, and washed with wash buffer containing D-PBS and 0.5% (w/v) BSA (PB buffer). This solution was centrifuged at 300 xg for 10 min at 4 °C, and the pellet resuspended in 90 μL of wash buffer and incubated with 10 μL of CD31-specific mouse microbeads for 15 min at 4 °C (#130–097–418, Miltenyi Biotech, Germany). After washing with 1 mL of PB buffer, the suspension was centrifuged at 300 xg for 5 min at 4 °C, the supernatant aspirated, and the pellet resuspended in 500 μL of PB buffer. The cell suspension was then transferred to MACS MS columns and subjected to magnetic separation using a MACS separator. CD31 positive cells were collected by washing the column with 500 μL of PB buffer and eluted into 900 μL of EBM-2 + [EBM-2 media supplemented with 2.5% (v/v) heat-inactivated FBS, 1% (v/v) penicillin–streptomycin, 10 mM HEPES and the following constituents of the EGM™−2 SingleQuot™ kit from Lonza (Lonza, Walkersville, MD): 0.01% (v/v) gentamicin-amphotericin, 0.01% (v/v) hydrocortisone, 0.01% (v/v) ascorbic acid, 0.025% (v/v) epidermal growth factor, 0.025% (v/v) insulin-like growth factor, 0.025% (v/v) vascular endothelial growth factor and 0.1% (v/v) basic fibroblast growth factor media]. The cell suspension was plated onto pre-collagenated 96 well plates (150 μL per well, 1% (v/v) Type I rat tail collagen in D-PBS) for cell viability assays or resuspended in 1.1 mL of EBM-2 + and subsequently plated onto pre-collagenated 6 well plates for protein abundance studies, mRNA quantification, and functional studies. Following plating, primary cells were incubated overnight at 37 °C, 5% CO2, 95% humidified air, and subjected to puromycin dihydrochloride treatment (3 μg/mL) for 72 h to select for endothelial cells, with media changes every two days. The above protocol has been validated by Fluorescence-Activated Cell Sorting (FACS) reporting a 91% purity of resulting endothelial cells [29].

### FAC Treatment of Primary MBECs and Viability Assessment

Primary MBECs were treated with FAC concentrations ranging from 1–250 µM for 48 h. *In vivo* models used to study iron overload report plasma and brain iron concentrations ranging between approximately 5 and 10 µM, with specific concentrations varying according to the dosage regimen applied [30, 31]. While higher than *in vivo* iron concentrations, the FAC concentrations used in this study align with previously reported concentrations effective for inducing iron overload *in vitro*,[27, 28, 32] with the shorter exposure of a higher concentration potentially reflecting longer term exposure expected *in vivo*. A FAC stock solution was freshly prepared in Milli-Q water, sterilized using a 0.22 µm filter, and diluted in EBM-2 + to the desired concentrations. Upon reaching ~ 80% confluency (as determined visually), MBECs were exposed to the FAC treatment solution and incubated at 37 °C, 5% CO_2_, and 95% humidity for 48 h. A 1% Milli-Q water in media vehicle control was included for all treatments to account for the fact that the FAC treatments included 1% Milli-Q water. Following treatment, MBECs were washed twice with D-PBS and incubated in 600 µL of MTT reagent (0.45 mg/mL MTT in EBM-2 basal medium with 10 mM HEPES, pH 7.4) at 37 °C, 5% CO_2_, and 95% humidity for 4 h. After incubation, the MTT reagent was replaced with 600 µL of DMSO per well and incubated at 37 °C for 30 min and absorbance was measured at 540 nm using a Perkin Elmer Multimode plate reader (Waltham, MA). Absorbance was adjusted for background by subtracting the reading of a blank well. Cell viability was expressed as a percentage of the absorbance of FAC-treated to vehicle-treated MBECs.

### Protein Isolation and Western Blot

Following treatment, MBECs were washed with 1 mL of ice-cold PBS and then 200 µL of freshly prepared lysis buffer was added to each well and incubated at 4 °C for 20 min with gentle agitation. The lysis buffer was made by combining 7 × protease inhibitor stock (1 Complete Mini Protease Inhibitor Cocktail tablet in 1.5 mL of Milli-Q water) and Pierce^®^IP lysis buffer in a 1:6 ratio. MBECs were scraped and transferred to pre-cooled Eppendorf tubes. Lysates were centrifuged at 14,000 xg for 10 min at 4 °C to separate cellular debris. The supernatant was aliquoted for protein quantification using a Pierce BCA protein assay kit (Thermo Scientific, Waltham, MA). For western blot analysis, 5 µg of MBEC lysate was combined with 6 × Laemmli buffer in a 5:1 ratio (0.125 M Tris–HCl, 10% (w/v) SDS, 5% (v/v) β-mercaptoethanol, 0.5% (w/v) bromophenol blue and 20% (v/v) glycerol)), vortexed, and centrifuged at 14,500 xg for 3 min. Samples were loaded onto either 4–15% or 4–20% acrylamide TGX™ gradient precast gels (Bio-Rad, Hercules, CA) along with 3 µL of Dual Xtra Precision Plus Protein Prestained Standards ladder (Bio-Rad). Electrophoresis was performed at 150 V for 1 h (P-gp/BCRP) or 200 V for 1 h (claudin-5) using a running buffer of 25 mM Tris base and 192 mM glycine. Proteins were transferred to a 0.22 µm nitrocellulose membrane using the Bio-Rad Trans-Blot^®^ Turbo™ apparatus. The membrane, gels, and the extra thick filter (blotting) paper were first equilibrated for 20 min in cold transfer buffer containing 25 mM Tris base, 192 mM glycine and 20% (v/v) methanol. The gel and membrane were then assembled in a ‘transfer stack’ between two pieces of the filter paper, secured into the apparatus and run at 25 V for 40 min (for P-gp), 25 V for 25 min (for BCRP) or 25 V for 42 min (for claudin-5). The membrane was rinsed with TBST (Tris buffered saline containing 0.05% (v/v) Tween-20), and total protein was quantified using Revert™ 700 Total Protein Stain Kits (LI-COR Biosciences, Lincoln, NE). The membrane was blocked with LI-COR Odyssey^®^ blocking buffer in PBS for 1.5 h at room temperature, followed by incubation at 4 °C overnight with one of the following primary antibodies diluted in TBST: mouse monoclonal C219 antibody for P-gp (#903701, 1:500, BioLegend, San Diego, CA), rabbit monoclonal primary antibody for BCRP (#EPR20080, 1:1000, Abcam), or rabbit monoclonal primary antibody for claudin-5 (#EPR7583, 1:10,000, Abcam, Cambridge, UK). Following overnight incubation, membranes were washed and incubated with one of the following secondary antibodies: IRDye^®^ 800CW goat anti-mouse IgG (1:7500, LI-COR Biosciences) for P-gp or donkey anti-rabbit 800LT IRDye^®^ (1:15,000, LI-COR Biosciences, Lincoln, NE) for BCRP /claudin-5. Proteins were detected at 800 nm using the Amersham™ Typhoon 5™ imaging system (GE Healthcare, Little Chalfont, Buckinghamshire, United Kingdom). Densitometric analysis was performed using Image J software (National Institutes of Health, Bethesda, MD), normalizing the signal to total protein in each lane. The fold-changes in protein abundance with FAC treatment were calculated as a ratio to the vehicle control treated MBECs.

### RNA Isolation and RT-qPCR

To assess alterations to *abcb1a* (P-gp), *abcg2* (BCRP), and *cldn5* (claudin-5) gene expression resulting from FAC treatments, MBECs were cultured in 6-well plates. Post-treatment, RNA was isolated using the RNeasy Mini Kit (Qiagen, Hilden, Germany) following the manufacturer's protocol. Media was aspirated, and wells were rinsed twice with ice-cold PBS. RNA lysate was prepared by adding 350 µL of RLT plus buffer containing 1% (v/v) β-mercaptoethanol per well, transferred to a QIAshredder column, and centrifuged at 14,000 xg for 2 min. Genomic DNA was removed by transferring the flow-through to a gDNA column and centrifuging at 14,000 xg for 1 min. The flow-through was mixed with 350 µL of 70% (v/v) ethanol, transferred to an RNeasy spin column, and centrifuged at 14,000 xg for 1 min. The mRNA was washed twice with 700 µL of buffer RW1 and 500 µL of buffer RPE. RNA was eluted by adding 50 µL of RNase-free water and centrifuging at 14,000 xg for 1 min. mRNA concentration was measured using a NanoDrop 1000 spectrophotometer (Thermo Fisher Scientific, Waltham, MA), with purity assessed by 260/280 nm absorption ratios (1.89–2.1) for RT-qPCR use. mRNA samples were diluted to 20 ng/µL in RNase-free water, and 5 µL of each sample was combined with 20 µL of PCR master mix ((12.5 µL 2 × RT-PCR reaction mix, 0.5 µL of iScript™ reserve transcriptase, 6.3 µL of nuclease-free water from the iTaq™ Universal Probes One-step kit (Bio-Rad, Hercules, CA)), and 0.7 µL of TaqMan^®^ probes and primer mix specific to the gene of interest [*abcb1a* (Assay ID: Mm00440761), *abcg2* (Assay ID: Mm00496364), *cldn5* (Assay ID: Mm00727012)], or housekeeping genes *gapdh* (Assay ID: Mm99999915) and *β-actin* (Assay ID: Hs: Mm02619580)] in a 96-well Thermowell Gold PCR plate (Corning, Corning, NY). RT-qPCR was performed using a Bio-Rad C1000 thermocycler in CFX96 system (Bio-Rad, Hercules, CA), with the following thermal cycling conditions: 50 °C for 10 min, 95 °C for 5 min, and 50 cycles of 95 °C for 15 s and 60 °C for 30 s. The relative gene expression was determined by the fold-change method ( 2^−∆∆*Ct*^) wherein the threshold cycles (C_t_) were automatically calculated by the CFX manager software, and the mRNA expression was normalized to gapdh and β-actin (housekeeping genes) [33]. The fold-change in mRNA in FAC-treated MBECs ( 2^−∆∆*Ct*^) was calculated as a ratio to the control-treated MBECs as per the following equations:

$$\begin{array}{c}\Delta {C}_{t}={C}_{t} \left(gene\;of\;interest\right)-\frac{{C}_{t} (\upbeta -actin)+{C}_{t} (gapdh)}{2}\\ \Delta \Delta {C}_{t}={C}_{t\left(treated\right)}-{C}_{t\left(control\right)}\\ {2}^{-\Delta \Delta Ct}=fold\;change\;in\;gene\;of\;interest\;normalised\;to\,\upbeta -actin\text{ and }gapdh\end{array}$$

### Measurement of P-gp, BCRP and Claudin-5 Function

To assess the effect of FAC-induced iron overload on P-gp, BCRP, and claudin-5 function, a polarized transwell model of primary MBECs was employed to evaluate bi-directional transport of ^3^H-digoxin (P-gp substrate), ^3^H-prazosin (BCRP substrate), and ^14^C-sucrose (paracellular permeability marker) [34–36]. MBECs were grown in 6-well plates to 80% confluency, then passaged onto Transwell (0.4 µm, polyester) inserts within a 24-well plate coated with 100 µL containing 40% (v/v) Type IV collagen, 10% v/v fibronectin and 50% v/v UltraPure™ distilled water [37]. Transendothelial electrical resistance (TEER) values were monitored daily using a MilliCell ERS-2 epithelial volt-Ohm meter (Merck Millipore, Burlington, MA) by placing electrodes in the upper and lower chambers. The output from the volt-Ohm meter represented the electrical resistance (Ω) of the MBEC monolayer (R_monolayer_) which was corrected for background using a media only Transwell insert (R_blank_). The final TEER value (Ω.cm^2^) was calculated using the following formula:

$$\mathrm{TEER}\;(\mathrm\Omega.\mathrm{cm}^2)=({\mathrm R}_{\mathrm{monolayer}}-{\mathrm R}_{\mathrm{blank}}) \,x\;\mathrm A\,(\mathrm{area}\;\mathrm{of}\;\mathrm{transwell}\;\mathrm{membrane}\;:\;0.33\;\mathrm{cm}^2)$$

TEER values were recorded daily and once TEER values plateaued, transwell media was replaced with Gibco™ human endothelial serum free medium (Life Technologies, Carlsbad, CA) supplemented with EGM™−2 SingleQuot™ kit, 1% (v/v) penicillin/streptomycin, 0.01% (v/v) hydrocortisone, and 0.5% (v/v) B-27™ supplement (Thermo Fisher Scientific, Waltham, MA) for 48 h to enhance barrier integrity. Incubation with Gibco™ human endothelial serum free medium was derived from the protocol for iPSC-derived EC Transwell studies [37]. A minimum TEER of 100 Ω.cm^2^ was considered indicative of a tight barrier. MBECs were then treated with FAC 250 µM or vehicle control for 48 h. Subsequent to treatment, MBECs were washed with blank EBM-2 media and incubated in EBM-2 + 10 mM HEPES (transport buffer) for 20 min. The donor chamber was then replaced with substrate solution [0.5 μCi] and immediately, an initial aliquot of the donor solution was taken to determine the initial donor chamber concentration of the compound. Aliquots were then taken from the receptor chamber at 5, 15, 30, 45 and 60 min, with an equivalent volume of fresh receptor solution added to maintain volume consistency, and concentrations were corrected to account for this dilution. Bi-directional transport was evaluated in abluminal (donor) to luminal (receptor) and luminal (donor) to abluminal (receptor) directions. Collected aliquots were combined with 2 mL of Ultima Gold™ scintillation mixture, and the radioactivity (DPM) was quantified using a Perkin-Elmer Tri-Carb 2800TR liquid scintillation counter (Boston, MA). DPM values were converted to Bq/mL, and using the manufacturer's specified specific activity (Bq/mmol), the resulting concentration (mmol/mL) was obtained. This was converted to mass (ng) using the molecular weight, and then to amount per well (ng/cm^2^). The bi-directional transport profiles of each substrate were represented as cumulative transport (% dose) *vs* time (minutes). The permeability coefficient (P_app_) of each compound was calculated using the following equation:

$${\mathrm P}_{\mathrm{app}}=(\mathrm{dQ}/\mathrm{dt})/({\mathrm C}_0\times\mathrm A)$$

where dQ/dt is the rate at which the compound appears in the receptor chamber measured from the linear portion of the bi-directional transport profile (i.e. cumulative transport *vs* time (minutes)), C_0_ is the concentration of compound in the donor chamber at time 0, and A is the cross-sectional area of the Transwell membrane (0.33 cm^2^).

### Assessing the Effect of Iron Overload on Brain Microvascular Abundance of Proteins In vivo

72 C57BL/6 mice (6–8 weeks old, male) were equally distributed into vehicle control (0.9% saline, *n* = 36) and iron treated (iron dextran (Sigma Aldrich, St. Louis, MO) 100 mg/kg, *n* = 36) groups. The mice were housed in groups of 5–6 per cage and were acclimatized for 7 days prior to beginning experiments. All mice had free access to food (standard chow, WEHI mouse breeder cubes, Ridley Agri Products, Melbourne, Victoria, Australia) and water throughout the housing period. According to a previous dosing regimen reported to increase plasma iron concentrations 3.9-fold without any toxicity, mice in the iron treated group were intraperitoneally administered 100 mg/kg of iron dextran, every 3 days for 4 weeks [38]. Mice in the vehicle group were intraperitoneally administered 0.9% saline every 3 days for 4 weeks. Three days after the last dose, all mice were processed for plasma and brain microvessel enriched fraction (MVEF) isolation. Mice were anesthetized with isoflurane, blood collected by cardiac puncture, and humanely killed via cervical dislocation. Brains were removed in a biosafety cabinet, with the cerebellum and occipital lobe excised and brains were pooled with those from five other mice to obtain sufficient protein. Pooled brains were homogenized in 10 mL of DMEM using a Dounce homogenizer (Tissue Grinder, Potter-ELV, Wheaton Industries, NJ) with 10 vertical strokes and 1–2 twists per stroke. The homogenate was transferred to a 50 mL falcon tube and centrifuged at 2000 xg at 4 °C for 5 min to obtain a pellet. The pellet was resuspended in 30% (w/v) BSA in DMEM (1:2 tissue ratio) and centrifuged at 2000 xg at 4 °C for 30 min to obtain three layers: 1) bottom pellet with capillary segments, 2) clear liquid layer, and 3) top cream layer with nervous tissue. Layers 2 and 3 were transferred to another tube and centrifuged again to obtain a second pellet of capillaries (capillary depleted fraction). Both pellets were combined and resuspended in 1 mL of DMEM and then filtered through a 100 µm mesh strainer to remove large blood vessels. The strainer was rinsed with DMEM (4 × 1 mL) and the filtrate centrifuged at 2000 xg at 4 °C for 5 min to obtain a pellet. The pellet was rinsed with 40 mL of PBS twice (centrifuged at 2000 xg at 4 °C for 5 min) and stored at −80 °C for future membrane fraction isolation. Membrane fractions of MVEFs were isolated using Mem-PER™ Plus Membrane Protein Extraction kit (Thermo Scientific, Waltham, MA). The cell pellet was thawed on ice and combined with 750 µL of cell permeabilization buffer, prepared by combining 50 mL of permeabilization buffer with 2 tablets of Complete mini protease inhibitor. The solution was transferred to a 1.5 mL Eppendorf tube and incubated at 4 °C for 10 min with constant mixing, and then centrifuged at 16,000 xg for 15 min at 4 °C. The supernatant containing cytosolic proteins was aliquoted, and the pellet was combined with 750 µL of membrane solubilization buffer (2 Complete Mini Protease Inhibitor Cocktail in 25 mL of Membrane Solubilization buffer). The solution was incubated at 4 °C for 30 min with constant mixing, then centrifuged at 16,000 xg for 15 min at 4 °C. The supernatant containing membrane fraction proteins was aliquoted for protein quantification assays using western blot methods identical to those previously described for MBECs in the methods section. The cytosolic fraction was used for ICP-MS.

### ICP-MS of Plasma and Brain MVEFs

The plasma, and cytosolic fractions of brain MVEFs isolated from both iron dextran (100 mg/kg) and vehicle-treated mice (*n* = 6–8) were lyophilized before sample preparation for ICP-MS analysis. Nitric acid (65% Suprapur, Merck, Darmstadt, Germany) was added to each sample and allowed to digest overnight. The samples were then further digested by heating at 90 °C for 20 min. An equivalent volume of 30% hydrogen peroxide (v/v) (Aristar, BDH) was subsequently added, with digestion continuing for 30 min. After allowing the samples to stop effervescing for 30 min, they were heated again at 70 °C for 15 min. The average reduction in volume was measured, and 50 μL aliquots were diluted 1:20 with 1% (v/v) nitric acid for the cytosolic fractions. ICP-MS measurements were conducted using an Agilent 7700 series ICP-MS instrument (Santa Clara, CA) under standard multi-element operating conditions with a Helium Reaction Gas Cell. The instrument was calibrated using certified multi-element ICP-MS standard solutions (0, 5, 10, 50, 100, and 500 ppb; Accustandard, New Haven, CT), and a 200-ppb certified standard solution of Yttrium (Y89) was used as an internal control. Elements included sodium, magnesium, phosphorus, calcium, iron, cobalt, nickel, copper, zinc, selenium, and rubidium. Elements with concentrations at or below detection limits were excluded from the final data analysis.

### Statistical and Graphical Analyses

Illustrations were created using BioRender (BioRender, Toronto, CA). All data analysis was performed using Graphpad Prism software, version 9 (GraphPad Software Incorporate, La Jolla, CA). When comparing data between two groups, an unpaired Student’s t-test was performed. When comparing data between more than two groups, a one-way analysis of variance (ANOVA) was performed, followed by a post hoc Dunnett’s multiple comparison test. When comparing two parameters at once, a two-way ANOVA followed by a post-hoc Tukey’s test was used to compare the mean of each group to all other groups. p values < 0.05 were considered statistically significant.

## Results

### Identification of Non-Toxic Concentrations of FAC in Primary MBECs

**Fig. 1:**
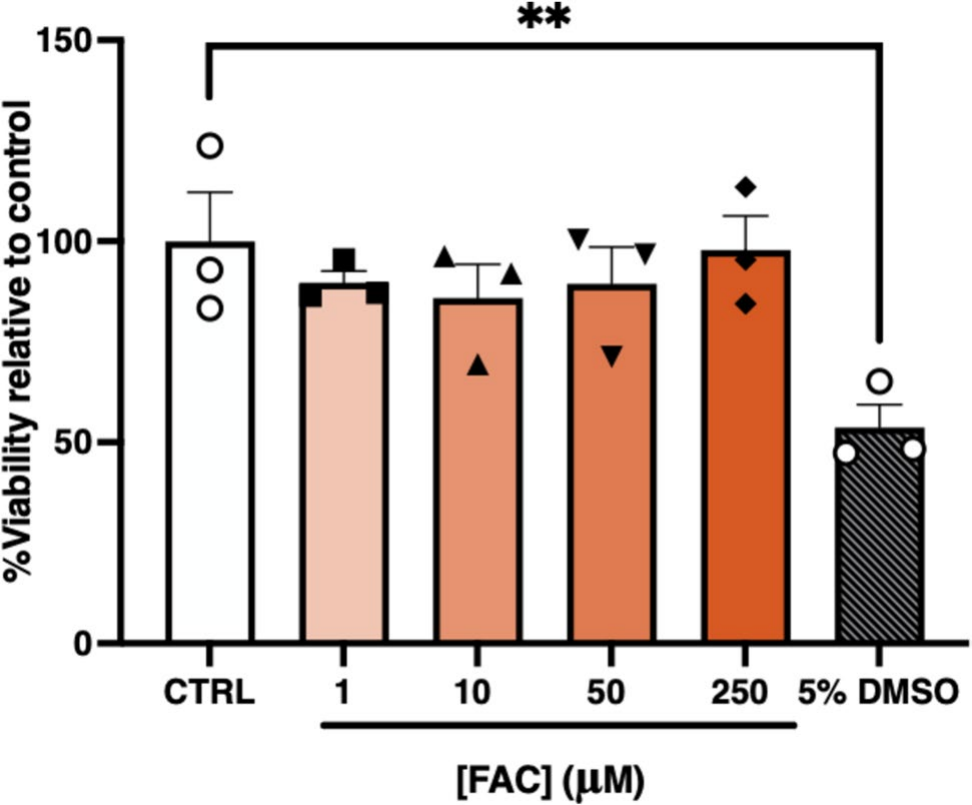
Viability of MBECs following a 48 h treatment with 1, 10, 50 or 250 µM of FAC. A vehicle control (CTRL), constituting 0.5% v/v Milli-Q water in media, was used and 5% v/v DMSO in media was included as a positive control for cell death. Data are presented as mean ± SEM (*n* = 3). **p < 0.01 relative to vehicle (CTRL) treated MBECs, as assessed by a one-way ANOVA followed by post-hoc Dunnett’s test.

As the impact of FAC on MBEC viability has not been previously studied, an MTT assay was undertaken to determine appropriate treatment concentrations. As shown in Fig. 1, FAC concentrations ranging between 1–250 µM did not negatively impact MBEC viability following a 48 h treatment. In contrast, the 5% DMSO treatment, used as a positive control for cell death, resulted in a significant reduction in cell viability by approximately 47% (*p* < 0.01). Given that FAC concentrations up to 250 µM did not negatively impact MBEC viability, this concentration was deemed appropriate for subsequent studies investigating the impact of iron overload on relevant barrier proteins in MBECs.

### FAC Differentially Modulates the Protein Abundance and mRNA Expression of Efflux Transporters and TJ Proteins in Primary MBECs

**Fig. 2:**
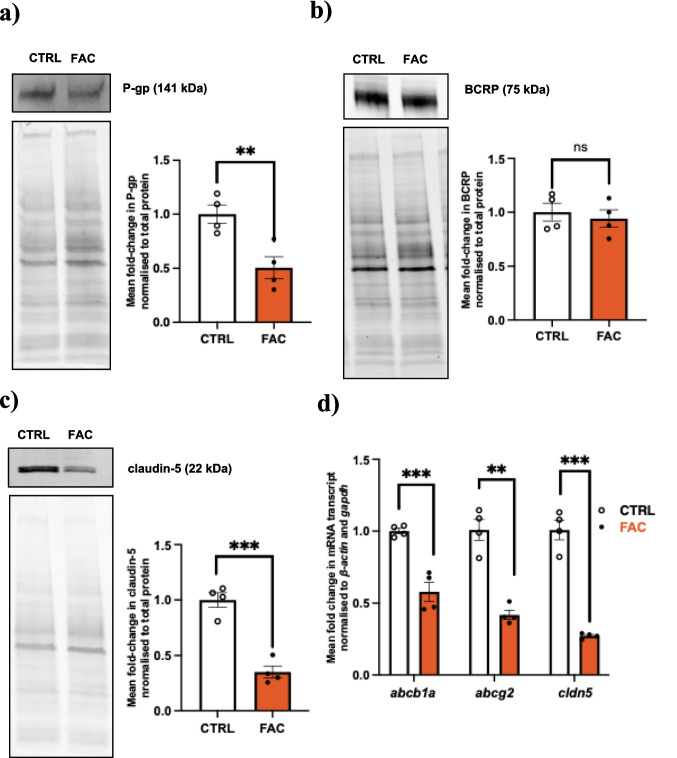
Representative western blots and total protein (left) and mean fold change in protein of interest normalized to total protein (right) demonstrating protein abundance in MBECs for (**a**) P-gp, (**b**) BCRP, and (**c**) claudin-5 and (**d**) mRNA transcripts of *abcb1a*, *abcg2* and *cldn5*, following treatment with 250 µM of FAC or vehicle (CTRL) for 48 h. Data are presented as mean ± SEM (*n* = 4) expressed as relative fold-change of respective CTRL, **p < 0.01, ***p < 0.001, and ns = not significant *p* > 0.05, when compared with CTRL as assessed by an unpaired Student’s t-test.

MBECs were treated with 250 µM of FAC for 48 h and assessed for protein abundance and mRNA transcript level changes via western blot and RT-qPCR, respectively. As shown in Fig. 2a, treatment with 250 µM of FAC resulted in a significant (*p* < 0.01) 50% reduction in P-gp protein compared to vehicle-treated MBECs. Correspondingly, a significant (*p* < 0.001) 43% reduction in *abcb1a* mRNA was observed (Fig. 2d), indicating a concurrent FAC induced downregulation at both the protein and transcriptional levels. Interestingly, while 250 µM of FAC did not elicit any significant changes to BCRP protein abundance (Fig. 2b), a significant (*p* < 0.01) 59% reduction in *abcg2* mRNA transcript was observed relative to vehicle-treated MBECs. In contrast, claudin-5 protein was significantly (*p* < 0.001) reduced by 65% following FAC treatment (Fig. 2c). Correspondingly, *cldn5* mRNA exhibited a significant (*p* < 0.001) 70% reduction following FAC treatment compared to vehicle-treated MBECs (Fig. 2d).

### FAC Does Not Alter P-gp or BCRP Function in MBECs

**Fig. 3:**
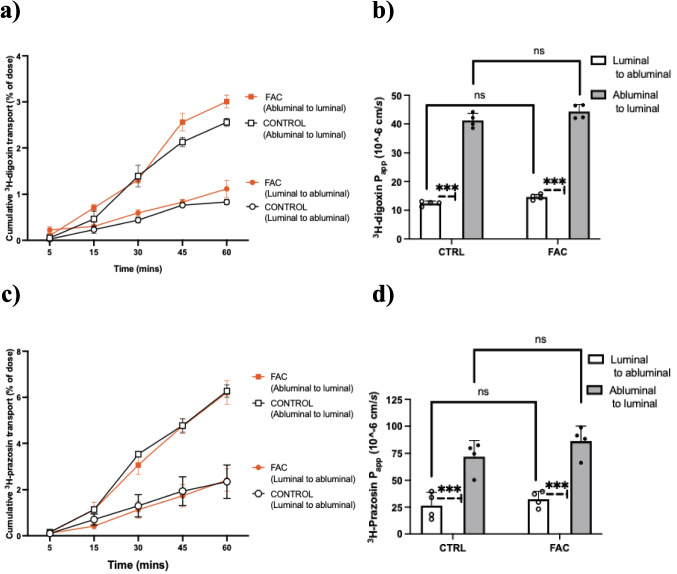
Bi-directional transport profiles (abluminal-to-luminal and luminal-to-abluminal) of (**a**) ^3^H-digoxin and (**c**) ^3^H-prazosin across MBECs treated with 250 µM of FAC or vehicle (CTRL) for 48 h. The luminal-to-abluminal and abluminal-to-luminal P_app_ values of (**b**) ^3^H-digoxin and (**d**) ^3^H-prazosin are presented. Data are presented as mean ± SEM (*n* = 4), ***p < 0.001 and ns = not significant when compared with vehicle (CTRL) treated MBECs, as assessed by a two-way ANOVA followed by post-hoc Tukey’s test.

The function of P-gp was assessed by measuring the bi-directional transport of the P-gp substrate ^3^H-digoxin. MBEC monolayers exhibiting a minimum TEER of 100 Ω.cm^2^ were utilized in bi-directional studies, with TEER values remaining similar before and after the experiment. The bi-directional transport profiles of ^3^H-digoxin across MBECs pre-treated with FAC or vehicle are shown in Fig. 3a. The transport of ^3^H-digoxin in both vehicle and 250 µM FAC-treated MBECs was polarized as shown by a significantly higher (*p* < 0.0001) abluminal-to-luminal P_app_ compared to luminal-to-abluminal P_app_ (Fig. 3b). Despite the FAC-induced reduction in P-gp protein abundance (Fig. 2a), the ^3^H-digoxin P_app_ was not significantly different between vehicle and FAC-treated MBECs in either direction (Fig. 3b), thereby demonstrating that FAC did not alter P-gp function.

Similarly, the effect of FAC on BCRP function was characterized by measuring the bi-directional transport of the BCRP substrate ^3^H-prazosin. The bi-directional transport profiles of ^3^H-prazosin across MBECs pre-treated with FAC or vehicle are demonstrated in Fig. 3c. In both vehicle and FAC-treated MBECs, ^3^H-prazosin exhibited polarized transport, indicated by a significantly higher (*p* < 0.001) abluminal-to-luminal P_app_ value compared to luminal-to-abluminal P_app_ value (Fig. 3d). Consistent with the lack of FAC-induced changes to BCRP protein (Fig. 2b), FAC did not impact BCRP function as no significant differences in ^3^H-prazosin P_app_ were observed between vehicle and FAC-treated MBECs in either direction (Fig. 3d).

### FAC Induced Downregulation of Claudin-5 is Associated with Increased MBEC Paracellular Permeability

**Fig. 4:**
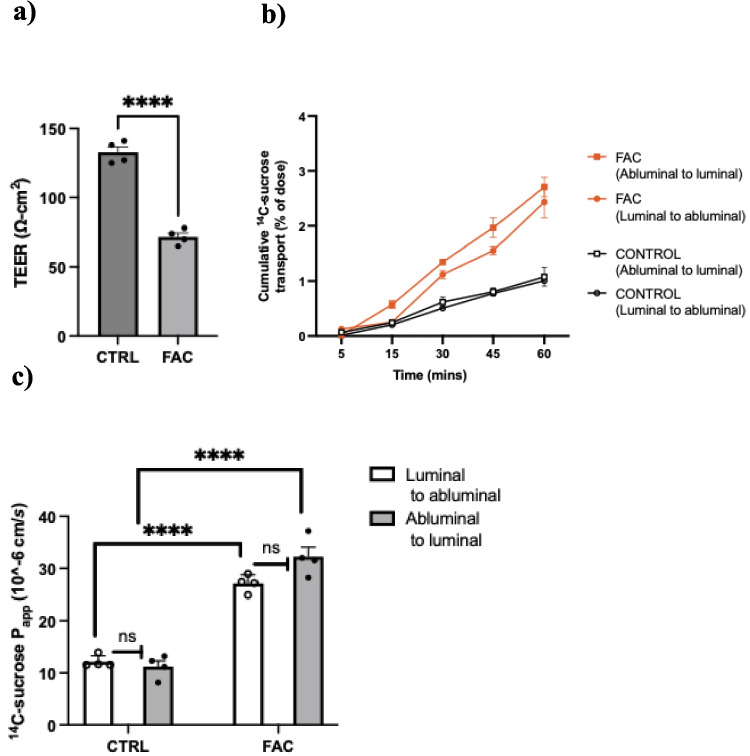
(**A**) Effect of FAC treatment on TEER values of MBECs, (**b**) bi-directional transport profiles (abluminal-to-luminal and luminal-to-abluminal) of ^14^C-sucrose in MBECs treated with 250 µM of FAC or vehicle (CTRL) for 48 h, and (**c**) the luminal-to-abluminal and abluminal-to-luminal P_app_ values of ^14^- sucrose are presented. Data are presented as mean ± SEM (*n* = 4), ****p < 0.0001 and ns = not significant when compared with vehicle-treated MBECs as assessed by an unpaired Student’s t test and, a two-way ANOVA followed by post-hoc Tukey’s test.

The bi-directional transport profiles of ^14^C-sucrose, as a marker of paracellular permeability, across MBECs treated with FAC or vehicle are shown in Fig. 4b. In both vehicle and FAC-treated MBECs, there was no significant difference between the luminal-to-abluminal and abluminal-to-luminal P_app_ values for ^14^C-sucrose, consistent with the expectation that paracellular permeability should be similar in both directions. The FAC treatment significantly increased paracellular permeability in MBECs, as demonstrated by a two-fold decrease in TEER values (Fig. 4a) and a corresponding significant increase (*p* < 0.0001) in P_app_ values of ^14^C-sucrose in both luminal-to-abluminal and abluminal-to-luminal directions compared to vehicle-treated MBECs (Fig. 4c). These changes align with a 65% reduction in claudin-5 protein abundance, indicating a loss of TJ integrity.

### Iron Dextran Does Not Modulate the Abundance of P-gp, BCRP and Claudin-5 in Brain Microvessel Enriched Fractions In Vivo

**Fig. 5:**
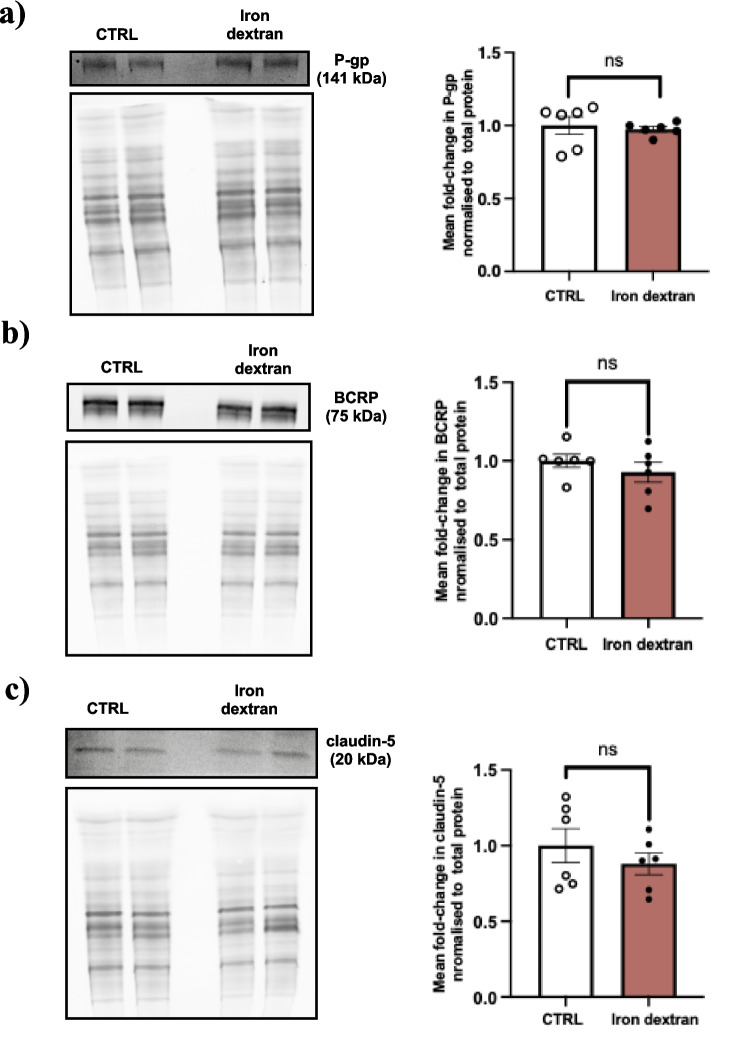
Representative western blots and total protein (left) and mean fold change in protein normalized to total protein (right) demonstrating abundance of (**a**) P-gp, (**b**) BCRP and (**c**) claudin-5 in membrane fractions of brain MVEFs obtained from C57BL/6 mice treated with 100 mg/kg treatment of iron dextran or vehicle (0.9% saline) (CTRL) every 3 days for 4 weeks. Data are presented as mean ± SEM (*n* = 6), ns = not significant when compared with vehicle treated mice, as assessed by an unpaired Student’s t-test.

Following a 4-week dosing regimen with iron dextran (100 mg/kg) or vehicle (0.9% saline) administered every 3 days, membrane fractions of brain MVEFs were isolated and assessed for protein abundance via western blot. Contrary to the differential effect of FAC on protein abundance in MBECs, the aforementioned iron-dextran dosing regimen did not elicit any changes to brain MVEF protein abundance of P-gp, BCRP, and claudin-5 *in vivo* (Fig. 5).

### Iron Dextran Increases Plasma Iron Concentrations Without Affecting Brain MVEF Iron Concentrations

**Fig. 6:**
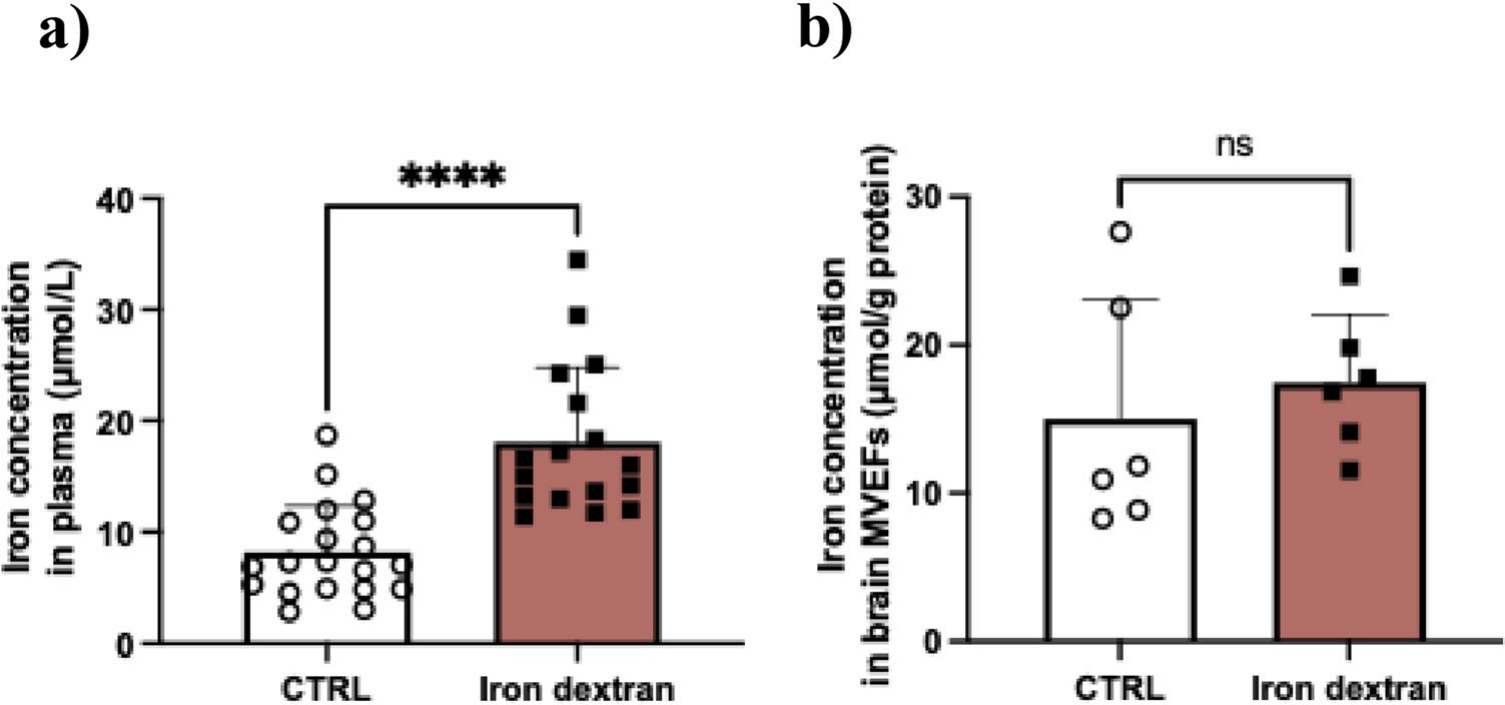
Iron concentration in a) plasma and b) cytosolic fraction of brain MVEFs following iron dextran administration. Mice were treated with 100 mg/kg of iron dextran or vehicle (0.9% saline) (CTRL) every 3 days for 4 weeks. Data are presented as mean ± SEM (*n* = 6–21), ****p < 0.0001 and ns = not significant when compared with vehicle-treated mice, as assessed by an unpaired Student’s t-test.

Iron concentrations in both mouse plasma and cytosolic fractions of brain MVEFs were evaluated using ICP-MS to ascertain potential relationships between MVEF iron concentrations and barrier protein abundance. The ICP-MS analysis revealed a significant 2.2-fold increase in plasma iron concentrations (Fig. 6a) in iron dextran-treated mice compared with vehicle-treated mice. Interestingly, despite this increase in plasma iron concentrations, there was no change in iron concentration in the cytosolic fraction of brain MVEFs as a result of iron dextran treatment (Fig. 6b), which correlated with a lack of change in barrier protein abundances (Fig. 5).

The analysis of metal concentrations, other than iron, showed no significant differences in brain MVEFs between the two treatment groups, but notable differences were observed in plasma (Table I). In plasma, iron dextran-treated mice exhibited a significant (*p* < 0.0001) 0.8–0.9-fold reduction in sodium, magnesium, phosphorus, and calcium. Conversely, there was a 1.6-fold increase in copper concentration in the plasma of iron dextran-treated mice.

**Table I:** Metallomic Analysis of Plasma and Cytosolic Fractions of Brain MVEFs Measured by ICP-MS. Concentrations of Metal ions in Plasma and Brain MVEFs Were Obtained from C57BL/6 Mice Following Treatment with Vehicle (0.9% saline) or Iron Dextran (100 mg/kg) Every 3 days for 4 weeks. Data are Presented as Mean ± SEM, with *p* < 0.05 Deemed Significantly Different to the Vehicle-Treated Group Assessed by an Unpaired t-Test. Fold Change Indicates the Relative Fold-Change in Iron Dextran-Treated Mice Compared to Vehicle-Treated Mice

Tissue	Element	Concentration in Vehicle Treated Mice (*n* = 6–21)	Concentration in Iron Dextran Treated Mice (*n* = 6–21)	*p* value	Fold Change if Significant
Plasma (µmol/L)	Sodium	19,388 ± 323.9	16,716 ± 673.1	0.0016	0.86
	Magnesium	118 ± 2.6	103 ± 5.6	0.0258	0.87
	Phosphorous	850 ± 25.2	708 ± 9	0.0014	0.83
	Potassium	525 ± 22.6	442 ± 23.1	0.2216	
	Calcium	297 ± 5.3	262 ± 11.1	0.0087	0.88
	Copper	15 ± 0.6	23 ± 2.2	0.0018	1.55
	Zinc	1.5 ± 0.03	1.5 ± 0.05	0.7751	
Microvessel Enriched Fraction (µmol/g protein)	Sodium	53,778 ± 6072	61,215 ± 4181	0.3368	
	Magnesium	199 ± 16.6	235 ± 5.5	0.0667	
	Phosphorous	3424 ± 465.3	3882 ± 222.8	0.3953	
	Potassium	2133 ± 240.5	2395 ± 58.3	0.3139	
	Calcium	1258 ± 142.1	1387 ± 193.8	0.6036	
	Copper	2.7 ± 0.3	2.6 ± 0.32	0.8070	
	Zinc	2.5 ± 0.3	2.7 ± 0.2	0.7168	

## Discussion

Iron overload and its concomitant oxidative damage have been shown to induce dopaminergic neuron toxicity and augmented amyloid-beta pathology, thereby playing a pivotal role in pathology associated with Alzheimer’s disease and Parkinson’s disease [39, 40]. Simultaneously, these diseases are afflicted with disruption of the BBB, wherein the reduction of TJ expression and efflux transporter dysregulation have been shown to contribute to altered brain homeostasis and increased neurotoxin levels [41, 42]. Previous studies using immortalized endothelial and epithelial cells have demonstrated that iron overload can induce downregulation of efflux proteins and reduce TJ expression [27, 28, 43]. Therefore, the aim of this study was to investigate the impact of iron overload on the expression and function of efflux transporters and TJs in primary MBECs and in mice, providing greater phenotypic complexity and physiological relevance compared to conventional immortalized cell models.

Based on an MTT cell viability assay, 250 µM of FAC over a 48 h treatment was deemed to be non-toxic to MBECs, and subsequently this intervention was used to induce iron overload *in vitro*. This concentration aligns with other studies that also utilize FAC to examine the impact of iron on efflux transporter levels in brain endothelial cells [27, 28]. Iron overload pathologies such as Alzheimer’s disease and Parkinson’s disease exhibit iron levels of approximately 12–20 µM in plasma, 0.1–0.5 µM in cerebrospinal fluid (CSF), and 5–10 µM in affected brain regions [44–47]. Additionally, our ICP-MS analysis from this study reports iron concentrations of 10–20 µM for plasma and 12–18 µmol/g for MVEFs, consistent with iron concentrations observed in Alzheimer’s disease transgenic mice [30]. Although our *in vitro* treatment concentration of 250 µM exceeds typical *in vivo* levels, this higher concentration was chosen to simulate chronic iron overload within a shorter timeframe—a common strategy used in studies modelling iron accumulation in neurodegenerative diseases [48, 49].

Our results demonstrated that treatment with 250 µM of FAC significantly reduced both P-gp protein and *abcb1a* mRNA, indicating a transcriptional regulation mechanism of FAC on P-gp abundance in MBECs. Interestingly, the magnitude of P-gp protein downregulation was larger than that observed with the reduction in mRNA, which is consistent with observations in immortalized human brain endothelial cells [27]. Since total cellular P-gp protein is influenced by transcriptional and post-transcriptional mechanisms, it is possible that FAC may have also stimulated P-gp degradation via pathways such as ubiquitin-proteasomal mediated degradation [50, 51]. Iron availability can indeed affect the ubiquitin-proteasomal system, potentially leading to increased degradation of P-gp proteins [52, 53]. To determine if FAC is also affecting P-gp degradation, MBECs could be treated with a proteasome inhibitor (e.g., MG132) in the presence and absence of FAC, and assess P-gp abundance over time, as has been reported in human colorectal cancer cells previously [54]. Aligning with a study conducted in immortalized hCMEC/D3 cells, our study demonstrates a differential effect of FAC on BCRP protein abundance and *abcg2* mRNA transcript [28]. Previous research in acute lymphoblastic leukaemia and acute myeloid leukaemia has shown a poor correlation between BCRP mRNA expression and protein abundance, which is aligned with our observations [55, 56]. Similarly, it is plausible that iron also stimulates BCRP protein degradation, [57] meaning that the overall impact of FAC on BCRP abundance is a result of reducing BCRP transcript and modulating protein clearance. Pertaining to the TJ protein claudin-5, FAC treatment induced a 65% downregulation in claudin-5 protein and a concurrent reduction in *cldn5* mRNA, thus suggesting that the effect of FAC on total protein was mediated through a transcriptional event. The pronounced downregulation of claudin-5 protein and mRNA suggests that iron overload might be rendering the barrier to be leaky, resulting in increased paracellular permeability [41]. This study is the first to demonstrate the specific effects of iron overload on claudin-5 contributing a possible link between the paracellular permeability dysfunction and brain iron accumulation in certain diseases such as Alzheimer’s disease and Parkinson’s disease [58, 59]. Acknowledging the role of hepcidin in regulating iron transport and the potential influence of citrate on its expression, it is important to address whether citrate, as part of the FAC composition, could contribute to the observed effects [60]. Previous studies in hCMEC/D3 cells demonstrated that treatment with ammonium sulfate and sodium citrate did not affect MDR1 or ABCG2 gene expression, suggesting that citrate alone does not play a regulatory role in these pathways [27, 28]. While these findings were observed in human brain endothelial cells, similar mechanisms regulating iron transport are likely present in mouse brain endothelial cells [61, 62]. Therefore, the changes in protein and mRNA abundance seen in our study are most likely driven by excess iron from FAC treatment rather than an effect of citrate alone.

Given our observations that FAC impacted the abundance of key proteins in MBECs, the bi-directional transport of substrates [^3^H-digoxin (P-gp), ^3^H-prazosin (BCRP) and ^14^C-sucrose (paracellular permeability marker)] was assessed. Both control and FAC treated MBECs demonstrated a polarized transport of ^3^H-digoxin and ^3^H-prazosin, as has been observed previously [36, 63]. Interestingly, discrepant with FAC induced downregulation of P-gp protein abundance, a concurrent reduction in P-gp function was not observed. Previous *in vitro* studies assessing P-gp function have reported that changes to P-gp abundance often correspond to less of a functional change [64, 65]. Thus, a possible explanation for the above inconsistency could be that the FAC induced 50% reduction in P-gp protein was insufficient to engender any changes to P-gp function. It is possible that even with a 50% reduction in protein levels, the remaining P-gp is still sufficient to maintain its function. It is also important to note, however, that the efflux activity of P-gp stems from membrane-bound P-gp whereas our studies assessed the abundance of P-gp in total MBEC lysate. Since we observed no change to P-gp function, it is possible that FAC might not be modifying membrane-bound P-gp and only cytosolic P-gp abundance. Although characterizing the impact of FAC on membrane bound P-gp could provide further mechanistic insights, this is not considered critical given the overall lack of functional changes in MBEC P-gp induced by FAC. Furthermore, the impact of FAC on BCRP function was also characterized, wherein consistent with the lack of changes to BCRP protein abundance in response to 250 µM of FAC, no change to BCRP function was observed. These findings underscore that despite the iron overload-induced downregulation of transporter proteins like P-gp and BCRP at the protein or mRNA level, their functional capacities can remain unaffected, likely due to complex regulatory mechanisms ensuring membrane localization and activity. In addition to the observations regarding P-gp and BCRP functionality, the impact of FAC on paracellular transport processes across a MBEC monolayer was assessed. Consistent with the pronounced 65% reduction in claudin-5 protein abundance, FAC treatment significantly increased the paracellular transport of ^14^C-sucrose in both abluminal-to-luminal and luminal-to-abluminal directions. Despite FAC-induced increases in paracellular permeability, ^3^H-digoxin and ^14^C-prazosin flux remained unchanged, likely due to their lipophilic nature and reliance on transcellular transport. Consistent with previous findings, disruptions to paracellular pathways do not always affect active transcellular processes [66], further supporting that these substrates are unaffected by changes to BBB paracellular function. It is important to note that this increase in permeability is unlikely due to a reduction in cell viability, as the viability study confirmed that FAC at 250 µM was non-toxic to the MBECs. These findings suggest that the BBB paracellular perturbation that has been observed in neurodegenerative diseases may, in part, be mediated by the iron overload in these diseases, although further studies are required to confirm this [58, 59].

Contrary to the *in vitro* results of the study, iron-dextran dosing to mice did not lead to changes to the membrane protein abundances of P-gp, BCRP and claudin-5. There are a number of potential reasons for this discrepancy between the *in vitro* and *in vivo* findings. In the *in vitro* study, primary MBECs were lysed at the end of the 48 h treatment, however the *in vivo* study involved isolation of brain MVEFs 3 days after the last dose of the 4-week dosing regimen. P-gp, BCRP and claudin-5 exhibit half-lives of 27 h, 35 h and 90 min, respectively [67–69]. Given the half-lives of P-gp, BCRP, and claudin-5, it is possible that transient changes in these proteins normalized during the 3-day delay between the last dose and the microvessel isolation, leading to the absence of detectable changes in this study. This is a plausible contributor to the observed findings given that Sripetchwandee *et al*. observed significant reductions in the tight junction protein occludin immediately after iron overload, highlighting the importance of timing in detecting these alterations [70]. To capture potential protein changes more accurately, future studies should consider a final timepoint closer to the culmination of the dosing regimen. Additionally, the use of microvascular-enriched fractions, which likely include cell types other than endothelial cells, such as vascular smooth muscle cells, could have influenced the measured protein abundance and iron levels, potentially confounding the results. Future studies using more refined isolation techniques to enrich for endothelial cells could help clarify these findings.

Secondly, the physicochemical properties of iron dextran may have also influenced *in vivo* results. Iron dextran, a large macromolecule unlikely to cross the BBB directly, relies on the slow release of iron into the plasma [71]. If this release is insufficient or results in forms of iron that have poor brain endothelial cell uptake, such as non–transferrin-bound iron, the iron available to brain endothelial cells may be limited [71, 72]. Compared to the *in vitro* experiments, which directly exposed cells to high concentrations of free iron using 250 µM of FAC, the *in vivo* dosing regimen may have resulted in lower iron exposure to the brain microvascular endothelial cells due to the need for iron to be released from iron dextran.

Finally, the concentration of iron available in the plasma to exert effects on brain microvascular barrier protein abundance is likely to contribute to the *in vitro*-*in vivo* discrepancy. While the ICP-MS revealed a significant 2.2-fold increase in iron concentrations in the plasma of iron dextran-treated mice compared to the control group, the concentrations of iron in plasma were approximately 14-fold lower than those used in the *in vitro* studies. Furthermore, no change in iron concentrations was observed in the isolated brain MVEFs. This stability in brain microvessel iron content likely reflects the protective function of the BBB, which is designed to prevent fluctuations in blood iron from translating changes to iron brain concentration. Taken together, the lower plasma concentrations of iron in the *in vivo* study (relative to *in vitro* studies) and the inability to increase brain MVEF concentrations of iron (likely due to sophisticated brain MVEF protective mechanisms) likely contributed significantly to the lack of effect of iron dextran on BBB barrier protein abundance. While the use of a higher iron dextran dose may yield the changes to brain MVEF barrier protein abundance, these studies should be carefully considered given the possibility of toxicity at higher doses. Therefore, probing the hypothesis that iron could modulate BBB trafficking processes *in vivo* may be evaluated with alternative methods of iron overload, such as genetic models of haemochromatosis which have been utilized in other studies [73]. Additionally, given that the function of the BBB is multifaceted involving a complex interplay of various proteins, it is important to acknowledge that evaluating BBB function through the expression of only three genes and proteins provides a limited perspective. Future studies could benefit from proteomic analyses to identify broader changes in barrier proteins and functional assays to assess specific pathways impacted by iron overload.

## Conclusion

Iron overload differentially modulated barrier proteins in MBECs with P-gp function remaining unchanged despite its downregulation and FAC mediating a downregulation of claudin-5 abundance and increased paracellular permeability. Iron dextran administration to mice increased plasma iron concentrations without affecting brain MVEF iron concentrations or abundance of key barrier proteins. These results suggest that the BBB modifications observed in neurodegenerative diseases with iron accumulation may in part, be mediated by iron overload, and highlight the importance of understanding the implications of iron overload on drug access to the brain.

## Declarations

Professor Ashley I. Bush has a profit share interest in, Collaborative Medicinal Development LLC and is a shareholder in Alterity Ltd., Cogstate Ltd. and Mesoblast Ltd.
